# Almen’s Test for Blood

**Published:** 1877-02

**Authors:** 


Almen's Test for Blood.—T. Schiellerup (Copenhagen) calls attention to the Almen's test, and warns against its use as being too delicate—one twenty-thousandth part of a milligram of iron (as chloride) being sufficient to produce the reaction. The test is as follows: A few cubic centimetres of tincture of guaiacum and an equal quantity of oil of turpentine are put into a test-tube, and a little of the suspected liquid (urine, etc.) added, when, in the presence of blood, an intense blue color is immediately produced; dried stains are extracted with diluted acetic acid (Proceedings Amer. Phar. Asso., 1875, p. 465). If one considers that iron is almost universally found, and that the reaction, as before mentioned, is so extremely delicate, it becomes evident that this test is a dangerous one in legal cases.—H. M. W. from *Ny Pharm. Tidskrift.*

